# A tale of two exemplars: the maternal and newborn mortality transitions of two state clusters in India

**DOI:** 10.1136/bmjgh-2022-011413

**Published:** 2024-05-02

**Authors:** Usha Ram, Banadakoppa Manjappa Ramesh, Andrea Katryn Blanchard, Kerry Scott, Prakash Kumar, Ritu Agrawal, Reynold Washington, Himanshu Bhushan, India Exemplars MNH study team, Mitali Raja

**Affiliations:** 1 Department of Bio-Statistics and Epidemiology, International Institute for Population Sciences, Mumbai, India; 2 Institute for Global Public Health, Department of Community Health Sciences, University of Manitoba Rady Faculty of Health Sciences, Winnipeg, Manitoba, Canada; 3 Johns Hopkins University Bloomberg School of Public Health, Baltimore, Maryland, USA; 4 India Health Action Trust, New Delhi, India; 5 National Health Systems Resource Centre, New Delhi, India

**Keywords:** Health policy, Health systems, Maternal health, Public Health, Child health

## Abstract

**Background:**

India’s progress in reducing maternal and newborn mortality since the 1990s has been exemplary across diverse contexts. This paper examines progress in two state clusters: higher mortality states (HMS) with lower per capita income and lower mortality states (LMS) with higher per capita income.

**Methods:**

We characterised state clusters’ progress in five characteristics of a mortality transition model (mortality levels, causes, health intervention coverage/equity, fertility and socioeconomic development) and examined health policy and systems changes. We conducted quantitative trend analyses, and qualitative document review, interviews and discussions with national and state experts.

**Results:**

Both clusters reduced maternal and neonatal mortality by over two-thirds and half respectively during 2000–2018. Neonatal deaths declined in HMS most on days 3–27, and in LMS on days 0–2. From 2005 to 2018, HMS improved coverage of antenatal care with contents (ANCq), institutional delivery and postnatal care (PNC) by over three-fold. In LMS, ANCq, institutional delivery and PNC rose by 1.4-fold. C-sections among the poorest increased from 1.5% to 7.1% in HMS and 5.6% to 19.4% in LMS.

Fewer high-risk births (to mothers <18 or 36+ years, birth interval <2 years, birth order 3+) contributed 15% and 6% to neonatal mortality decline in HMS and LMS, respectively. Socioeconomic development improved in both clusters between 2005 and 2021; HMS saw more rapid increases than LMS in women’s literacy (1.5-fold), household electricity (by 2-fold), improved sanitation (3.2-fold) and telephone access (6-fold).

India’s National (Rural) Health Mission’s financial and administrative flexibility allowed states to tailor health system reforms. HMS expanded public health resources and financial schemes, while LMS further improved care at hospitals and among the poorest.

**Conclusion:**

Two state clusters in India progressed in different mortality transitions, with efforts to maximise coverage at increasingly advanced levels of healthcare, alongside socioeconomic improvements. The transition model characterises progress and guides further advances in maternal and newborn survival.

**WHAT IS ALREADY KNOWN ON THIS TOPIC**

- India’s national progress in reducing maternal and neonatal mortality since 2000 is attributable to improvements across states with different mortality levels, health systems and socioeconomic environments. While most studies have analysed broad trends, none have systematically examined and compared the interrelated factors marking mortality reduction transitions in India’s states starting both at higher and lower baseline levels.

**WHAT THIS STUDY ADDS**

- This study employs a unique approach to comprehensively assess and analyse how neonatal mortality rate and maternal mortality ratio reduction has been achieved by state clusters starting from higher and lower mortality levels within a common national policy landscape. In doing so, it illustrates the transition model’s value for understanding how to reduce mortality at different stages in the pursuit of the Sustainable Development Goals.

**HOW THIS STUDY MIGHT AFFECT RESEARCH, PRACTICE OR POLICY**

- The study’s use of a maternal and neonatal mortality transition model helps benchmark the journeys of two state clusters. The demographic, socioeconomic, and health policy and systems pathways marking the clusters’ transitions can provide guidance for contexts currently at higher maternal and neonatal mortality levels seeking to make further progress. It showcases a comparative learning model that is evidence based, strengths based, sustainable and focused on equity.

## Background

 India is one of the world’s most diverse countries. Its 1.3 billion people, spread across 28 states and 8 union territories, showcase great ethnic, historical, cultural and demographic heterogeneity. States have followed varied trajectories in their socioeconomic development, demographic changes and health outcomes, depending on their sociocultural and historical contexts, and direction of the respective state governments within India’s federal governance structure.^1 2^

India’s progress in improving maternal and newborn health (MNH) over the past two decades has been exemplary, outpacing global and regional declines with or without adjustment for economic growth.^3 4^ During 2000–2018, the national maternal mortality ratio (MMR) dropped from 327 to 103 per 100 000 live births, and neonatal mortality rate (NMR) from 44 to 23 per 1000 live births.^5 6^ Yet it is important to understand how the improvements in MNH have followed different trajectories and momentum in the heterogeneous states and regions. For instance, in 1999–2001, the MMR ranged from 149 per 1000 live births in Kerala to 539 in Uttar Pradesh. Both these states achieved massive reductions in MMR by 2016–2018 to 43 and 197, respectively, but at different speeds. Similarly, in 2000, the NMR ranged from 9.8 per 1000 live births in Kerala to 61.1 in Odisha, followed by a massive NMR reduction in both these states, at almost equal speeds.

To characterise, compare and understand states’ different mortality transitions over the past few decades, we identified two clusters of states using empirical data on mortality trends by per capita income (PCI). We adapted a five-stage maternal and neonatal mortality transition model developed as part of the global Exemplars in maternal and neonatal mortality reduction study, to examine the state clusters’ transition in five key characteristics: mortality levels, timing and cause of death patterns, health intervention coverage and equity, fertility, and socioeconomic changes.^7^ Additionally, we assessed how India’s major health policy and systems changes since the 1990s relate to state clusters’ mortality transitions. Guided by the transition model, this study interprets past trends and assesses the current situation in the transition characteristics to help inform future strategies for India’s states and other regions aiming to reach advanced stages of mortality reduction.^7^

## Methods

### State clusters

**Figure 1:**
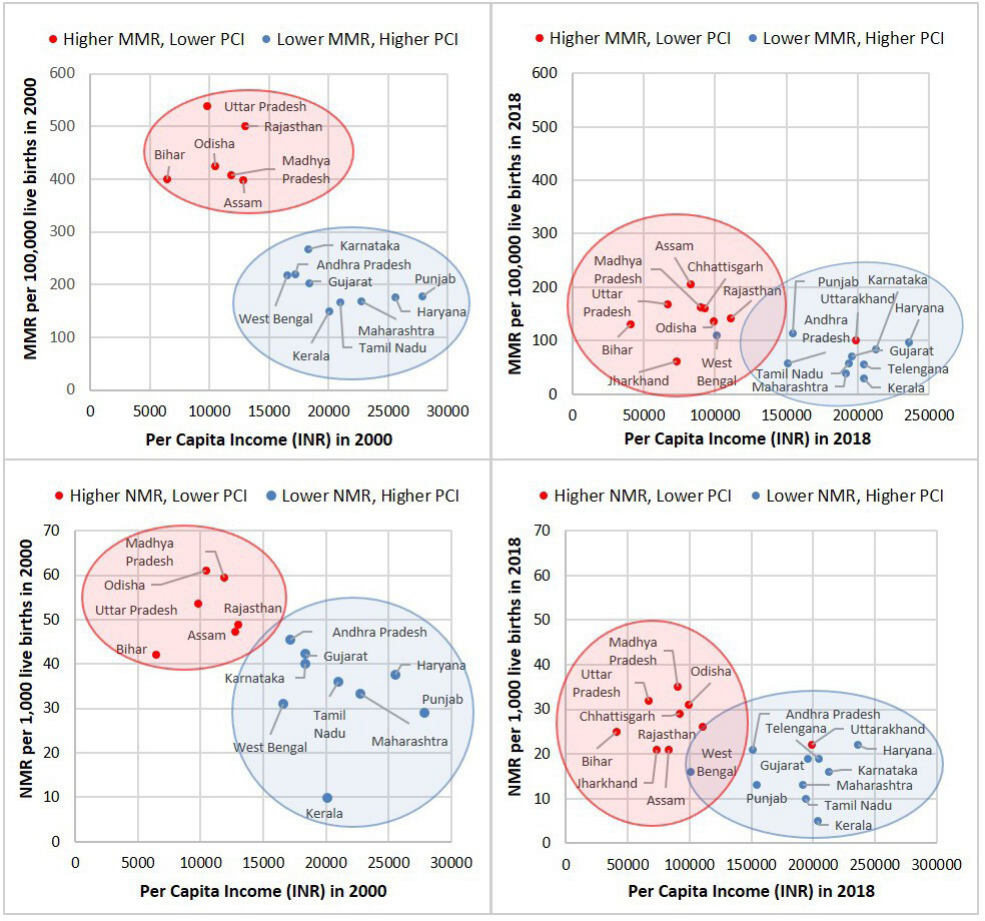
Comparison of state-specific maternal mortality ratio (MMR) and neonatal mortality rate (NMR) levels in 2000 and 2018 by state per capita income (PCI) (SRS). West Bengal with a similar MMR and NMR as lower mortality state (LMS) yet lower per capita income in 2018 was included in the LMS cluster. Uttarakhand with a similar MMR and NMR to higher mortality state (HMS), but higher PCI in 2018 was included in the HMS cluster. In 2000, Uttarakhand was part of Uttar Pradesh, Telangana was part of Andhra Pradesh and Jharkhand was part of Chhattisgarh, but by 2018, they were all separate states.

We mapped all major Indian states according to their mortality rates and PCI in 2000 and 2018. The states clustered into two clear groupings, as shown in figure 1: one of higher mortality states (HMS) with lower PCI and one of lower mortality states (LMS) with higher PCI. The two state clusters resulting from this approach include the following (online supplemental figure S1):

- HMS with lower PCI (49% of India’s population): Bihar, Chhattisgarh, Jharkhand, Madhya Pradesh, Odisha, Rajasthan, Uttar Pradesh, Uttarakhand (comprising the Empowered Action Group states^8^) and Assam.
- LMS with higher PCI (47% of India’s population): Andhra Pradesh, Gujarat, Haryana, Karnataka, Kerala, Maharashtra, Punjab, Tamil Nadu, Telangana and West Bengal.

### Mortality transition model stages

The global MNH Exemplars study developed a five-stage integrated framework for a maternal, stillbirth and neonatal mortality transition using empirical trends from nearly 150 countries on the following related characteristics: levels, timing and cause of death patterns, intervention coverage and equity, fertility and socioeconomic development (such as household conditions and women’s education).^7^ It posits a shift from increasing intervention coverage for basic MNH services, towards increasingly advanced MNH services offered at hospitals, more than lower-level facilities, and particularly facilities with the capacity to perform Caesarean or C-sections. Moving across stages, these shifts in health service coverage are expected to be catalysed by intentional health policies and systems efforts (online supplemental table S1). Unlike the global model that includes stillbirths, because we found that stillbirth data in India suffer from major underestimation, this analysis of India’s two state clusters is focused on transitions in maternal and neonatal mortality.

### Study design

An iterative, mixed-methods approach was used to understand India’s exemplary maternal and newborn mortality transitions. We quantitatively described mortality trends in the two state clusters. We used available quantitative data to analyse related changes in mortality timing and causes, intervention coverage overall and by facility level, coverage inequalities, fertility changes and socioeconomic development. Concurrently, we analysed related health policy and systems changes using literature, documents and qualitative key informant interviews (KIIs) and state and national-level expert round table discussions.

To aid our analyses, we identified four national policy periods relevant to MNH in India: the Child Survival and Safe Motherhood Programme (CSSM) from 1992 to 1997, Reproductive and Child Health I (RCH I) from 1997 to 2005, RCH II and the National Rural Health Mission (NRHM) from 2005 to 2012 and the Reproductive, Maternal, Neonatal, Child and Adolescent Health programme and National Health Mission (NHM) from 2012 to 2020.

### Data sources

We used the Sample Registration System (SRS) for maternal and neonatal mortality and fertility trends. The national household surveys including the National Family Health Survey (NFHS, five rounds),and the District Level Household Survey (DLHS, three rounds) were pooled for the trends in intervention coverage and equity. For causes of death trends, we used the Million Death Study (MDS) for 2005/2006^9 10^ and reviewed estimates from the World Health Organization/Maternal and Child Epidemiology Estimation (WHO/MCEE)^11^ and the Global Burden of Disease (GBD) study.^12^

For the qualitative component, we organised a national stakeholder meeting (length: 2 hours and 10 min) with 13 experts in June 2021 to identify key drivers of mortality decline. KIIs averaged 1.5 hours and were conducted from July to November 2021. We invited 21 experts active since 2000 in MNH policy and implementation from the government, donor organisations, private, civil society and academic spheres, of which 13 consented. We selected six states for in-depth analysis with the highest combined average annual rates of change (AARC) for MMR and NMR since 2000 (Maharashtra and Tamil Nadu from the LMS cluster; Rajasthan, Odisha, Uttar Pradesh and Madhya Pradesh from the HMS cluster) and held one round table discussion with state-level experts in the six states separately (n=11 each on average) in March–April 2022, to identify key policy and health system drivers of mortality declines (averaging 3.15 hours). All were conducted on Zoom in English, audio recorded and transcribed.

### Analytical methods

We analysed quantitative trends by computing AARCs through regression analysis^13^ for the different national policy periods. To measure ANC with contents and intensity-related components, we computed a composite index called ANCq,^14^ which has a 13-point scale. After adaptation to India, our ANCq index consisted of the number of ANC visits, timing of ANC, at least one ANC by a skilled provider, blood pressure checked, weight measured, abdomen examined, blood sample collected, urine sample collected and the number of tetanus toxoid vaccinations during pregnancy.

We analysed institutional delivery rates overall and at public (district) hospitals, private hospitals, or lower-level facilities (community health centres or primary health centres, with variable capacity for emergency care, and fewer in-patient beds than hospitals).

For measuring equity in intervention coverage, we used the absolute slope index of inequality (SII) multiplied by 100, interpreted as the percentage point difference in coverage between the most and least disadvantaged.^15^ Household wealth tertiles are used instead of quintiles to ensure larger sample sizes particularly in NFHS 1998–1999 and 2005–2006.

We conducted bivariate decomposition analyses in STATA version 16.1^16^ to measure the relative contribution of compositional changes in terms of high-risk birth categories (eg, in single or multiple high-risk categories) and changes in relative risk (eg, faster decline in mortality among births in any high-risk category) to NMR reduction. High-risk birth categories included: mother aged less than 18 or over 35 years, previous birth interval under two years, and a birth order of over three. The first order births not in any high-risk category were grouped into the ‘unavoidable risk category’. We also applied the Jain’s method of decomposition^17^ on the SRS data to analyse the contribution of fertility decline to reductions in NMR and MMR as well as the maternal and newborn lives saved in the two state clusters.

We coded the qualitative transcripts in Dedoose software using a codebook developed based on *a priori* topics, and additional emergent subcodes. We shared synthesised results with key informants anonymously to finalise the results.

### Patient and public involvement

No patients were involved in this study. The study was coordinated by the National Health Systems Resource Centre, a Government of India technical support centre, in collaboration with the International Institute for Population Sciences (Mumbai), supported by the India Health Action Trust (New Delhi) and the University of Manitoba, Canada (online supplemental materials 2). Expert stakeholder meetings were used to disseminate the quantitative findings, and informed subsequent in-depth policy analysis and KIIs. Those results were further disseminated to participants for additional information and feedback.

## Results

### Trends in maternal and neonatal mortality

**Figure 2:**
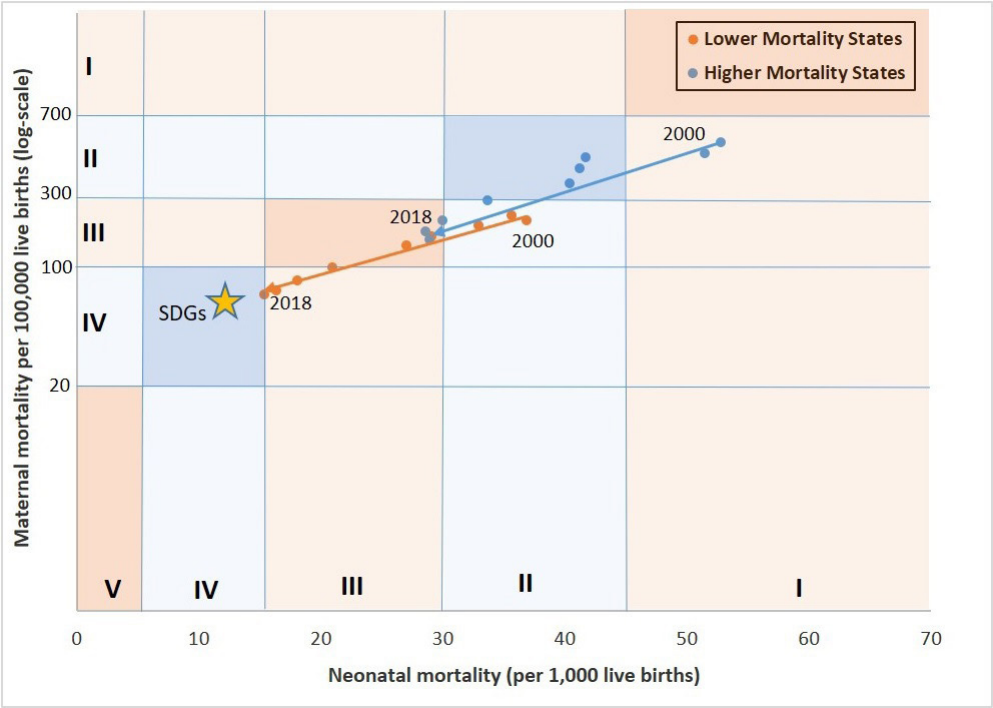
Mortality transition in India’s lower and higher mortality state clusters (SRS, 2000–2018). The dots represent the mortality estimates published by SRS from 2000 to 2018. The solid lines connect the 2000 SRS estimates to the 2018 estimates for the two clusters separately.

While the clusters started at higher and lower baseline levels, both showed remarkable mortality rate declines moving across MNH transition stages in the past two decades (figure 2). From 2000 to 2018, HMS transitioned from stages I to III, with a 69% reduction in MMR and a 44% reduction in NMR. In that time, LMS transitioned from stages II to IV, reducing MMR by 65% and NMR by 57%. The LMS have a 12-year and 16-year lead ahead of the HMS in maternal and neonatal mortality transitions, respectively.

**Figure 3:**
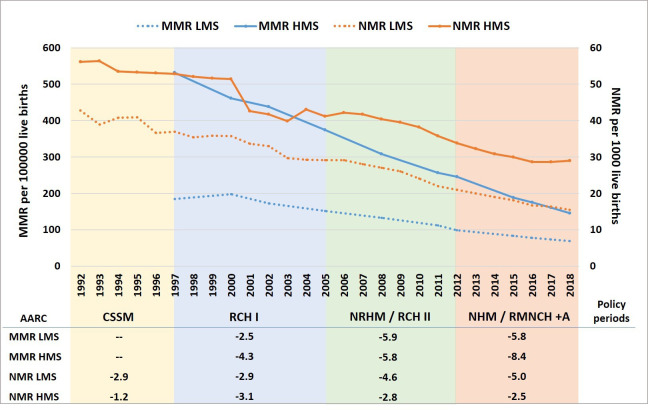
Neonatal mortality rate (NMR) (3-year moving averages) and maternal mortality ratio (MMR) trends, and the average annual rates of change (AARC, %) in different policy periods, in lower and higher mortality state clusters (SRS, 1992–2018). CSSM, Child Survival and Safe Motherhood; NHM, National Health Mission; NRHM, National Rural Health Mission; RCH I, Reproductive and Child Health I; SRS, Sample Registration System.

MMR declined most rapidly in both clusters in the NRHM and NHM periods. HMS’ MMR declined from nearly 400 in 2005 to 145 in 2018, the level among LMS in 2005 (figure 3). From an MMR of 151 in 2005, it took LMS 13 years to reach the Sustainable Development Goals (SDG) 2030 target of 70 in 2018. If HMS achieve a similar pace of decline as LMS, they will be in early mortality stage IV by 2035 and approach the SDG targets.

The NMR in HMS declined from 52 to 29 per 1000 live births, and in LMS from 36 to 15 per 1000 live births during 2000–2018. The lag between clusters in achieving an NMR of 30 per 1000 live births was 12 years (2003 in LMS and 2015 in HMS). The pace of the NMR decline was fastest in LMS after 2005, during the NRHM and NHM policy periods. If the same pace continues, LMS may reach the SDG target of 12 much before 2030. HMS also maintained a consistent decline in NMR since the late 1990s. If HMS reach a similar pace of change as LMS have, the SDG NMR target will be nearly reached by 2030.

While both 0–2 days and 3–27 days mortality rates declined in both clusters, the decline in 0–2 days mortality was much faster in LMS than HMS (online supplemental figure S2). While the 0–2 days mortality in LMS accelerated during the NRHM/RCH-II period (2005–2012), it started to decline in HMS only in the most recent NHM policy period of 2012–2018. About 60% of the NMR decline in LMS during 2005–2018 was due to declines in deaths on days 0–2, while this proportion was less than 30% in HMS.

### Causes of maternal and neonatal mortality

Though insufficient to consistently quantify trends, available data indicate similar leading causes of maternal death in each cluster: haemorrhage (mostly postpartum), pregnancy-related infections, hypertensive disorders of pregnancy, abortion-related complications, obstructed labour or other complications of labour and delivery. Indirect causes (such as anaemia, malaria, diabetes or heart disease) accounted for an increasing share of the deaths especially in the LMS as levels of MMR declined.^18^

Data from the MDS and global estimates for 2000 and 2015–2019 on causes of neonatal death indicate a decrease in the relative importance of infectious diseases and to a lesser extent the peripartum complications, and an increase in causes related to the health and nutritional status of women and babies in both state clusters (online supplemental figure S3). The main difference between the clusters according to MDS (2000–2015) and India GBD study data (2000–2017) was that neonatal deaths due to preterm birth-related complications declined more rapidly in LMS than HMS.

### Coverage of MNH interventions across continuum of care

**Figure 4:**
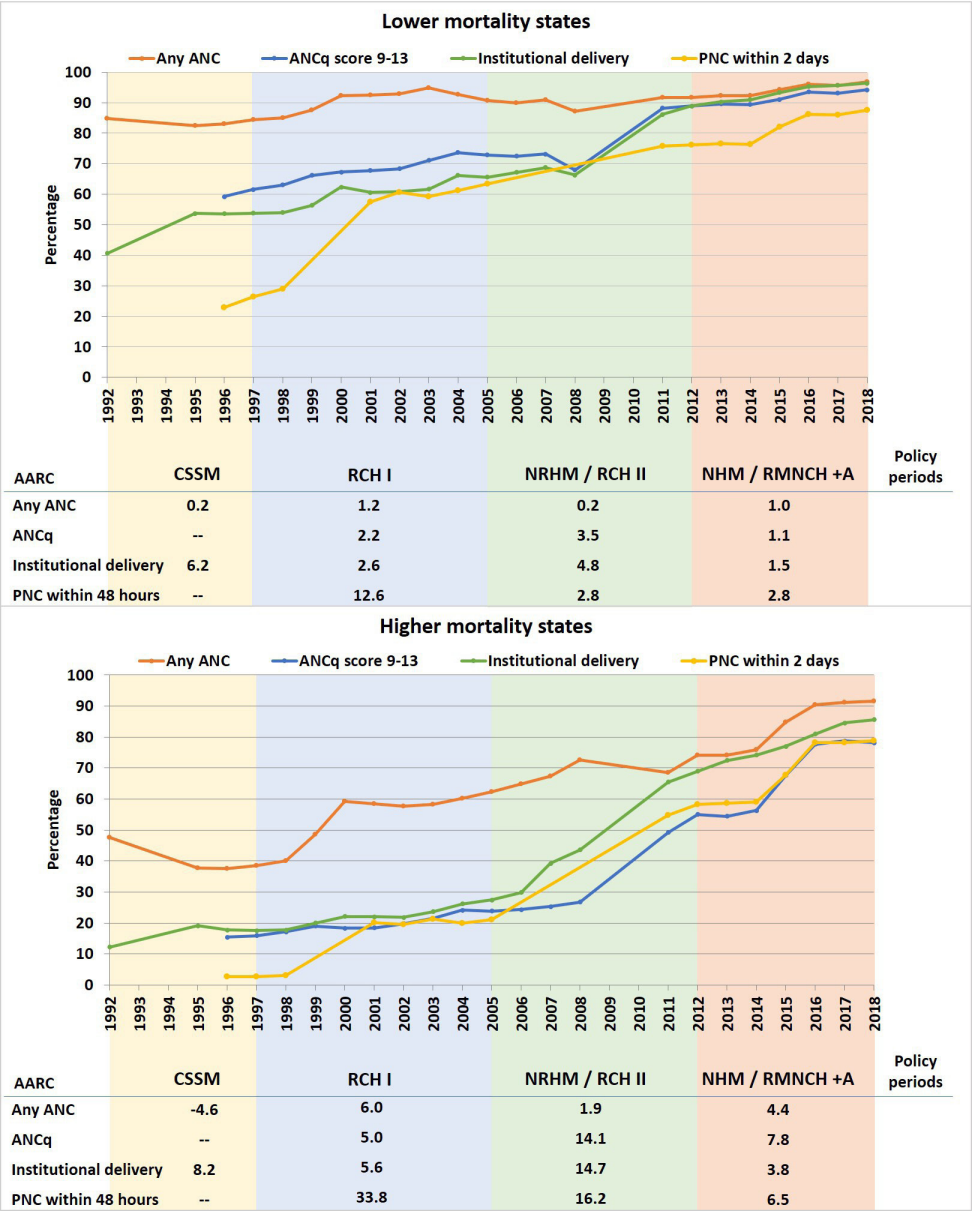
Trends in any antenatal care (ANC), antenatal care with contents (ANCq), institutional delivery and postnatal care (PNC) within 48 hours of birth (%), with average annual rates of change (AARC, %) in different policy periods, in the two state clusters (NFHS and DLHS pooled data, 1992–2018). CSSM, Child Survival and Safe Motherhood; DLHS, District Level Household Survey; NFHS, National Family Health Survey; NHM, National Health Mission; NRHM, National Rural Health Mission; RMNCH+A, Reproductive, Maternal, Neonatal, Child and Adolescent Health.

All four intervention coverage indicators improved in both clusters throughout the study period (figure 4). In LMS, the increase was gradual from a higher baseline, with ANC, ANCq and institutional delivery reaching about 90% by 2013, and PNC within 2 days reaching close to 90% in 2018. In HMS, there was a prolonged and steep increase from low rates in the early 2000s, reaching 79%–92% by 2018. The coverage for institutional delivery increased fastest in both of the clusters in the RCH-II/NRHM period of 2005–2012, with an AARC in HMS triple that of LMS.

Institutional delivery in LMS increased most at hospitals compared with lower-level facilities. This increase in hospital deliveries was driven first by a steady increase in private hospitals and then, from 2008, by an increase in public hospital deliveries (online supplemental figure S4). In HMS, however, the increase in institutional delivery was driven mainly by increasing lower-level public facility deliveries. Comparatively, HMS’ hospital deliveries increased from below 20% in 2006 up to 40% in 2018 overall (equivalent proportions in public and private sectors), which was reached by LMS in 1998. In 2018, there was a two-fold difference in lower-level facility deliveries between the two clusters (46% in HMS and 23% in LMS).

In LMS, C-section rates increased rapidly from 2008 to 2011 from 13% to 25%, driven by increases in both private and public sectors. C-section rates also increased in HMS, from 5% in 2008 to 14% in 2018, a few years after the major increases in institutional birth rates. State clusters’ differential C-section rates were more pronounced among the poorest than overall in 2018: 20% in LMS but only 7% in HMS, indicating persistent unmet need among the poorest in HMS (online supplemental figure S5).

### Inequalities in coverage

**Figure 5:**
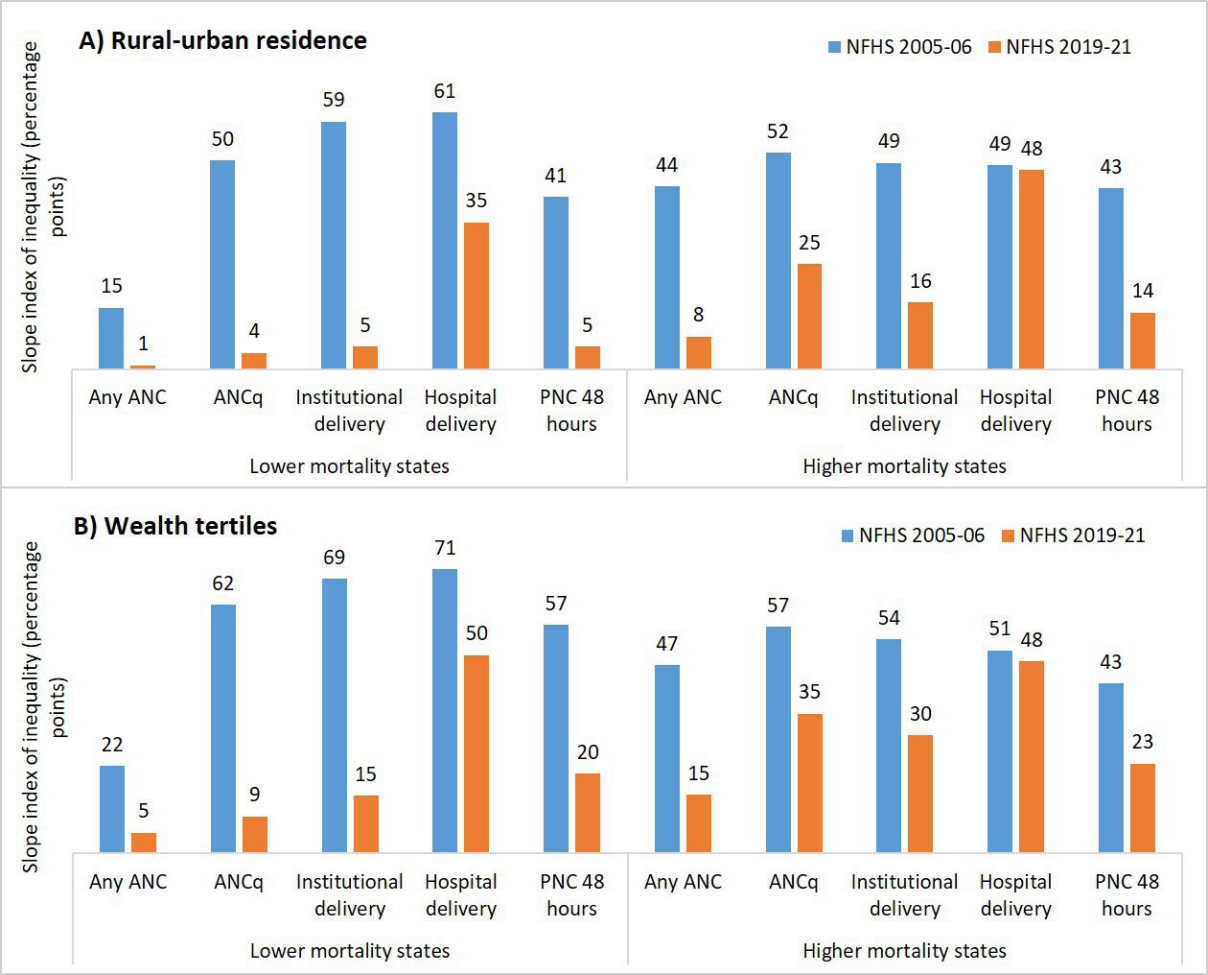
Trends in coverage inequalities in the two state clusters between A) urban/rural residence and B) wealth tertiles using slope index of inequality (percentage points) (NFHS, 2005-6, 2019-21). DLHS, District Level Household Survey; NFHS, National Family Health Survey.

Coverage inequalities by urban/rural residence and wealth tertile reduced considerably in both clusters for all interventions, as measured by the SII. The only exception was hospital deliveries in HMS, in which inequalities did not change (figure 5A,B). Inequalities in hospital deliveries in LMS reduced but remained high, at 35 percentage points between urban and rural areas, and 50 percentage points between rich and poor.

### Contribution of fertility changes

Fertility has steadily declined in both state clusters since the 1970s (online supplemental figure S6). HMS’ total fertility rate (TFR) was higher but declined from 4.7 in 1990 to 4.2 in 2000, and 2.6 in 2019. In LMS, TFR was already 3.0 in 1990, declining to 2.4 in 2000 and under 1.6 in 2019. The clusters’ differential narrowed over time between HMS and LMS, from 1.8 children in 2000 to 1.0 in 2019. Although fertility declined in both the clusters, population momentum was different. The number of births in HMS increased from 14.6 to 15.8 million between 2000 and 2019, while it reduced from 10.5 to 9.8 million in LMS.

With declining fertility, the risk profile of births changed in the two state clusters (online supplemental figure S7). The proportion of births among women in any avoidable risk category was higher in HMS (54%) than LMS (35%) and declined at a similar pace since 2005–2006. Bivariate decomposition analysis indicated that compositional changes in births from fertility reduction contributed 13% and 7% of the overall NMR reduction in HMS and LMS, respectively, during this period (data not shown). Jain decomposition method resulted in somewhat larger estimates of the contribution of fertility decline to maternal and neonatal mortality declines in the two state clusters (online supplemental table S2).

### Socioeconomic development

**Figure 6:**
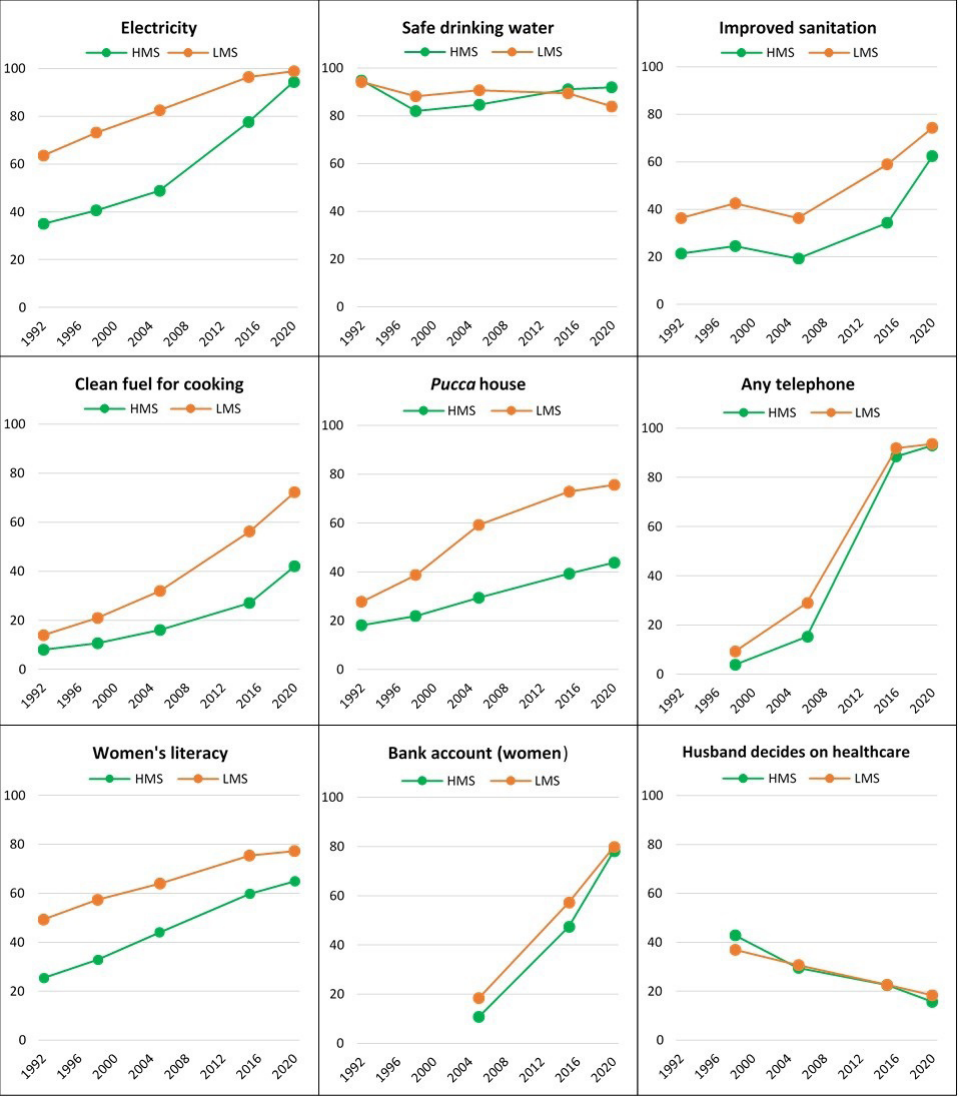
Trends in socioeconomic development indicators in the two state clusters (NFHS, 1992-3, 1998-99, 2005-06, 2015-16 & 2019-2021). HMS, higher mortality state; LMS, lower mortality state; NFHS, National Family Health Survey.

The proportion of households with electricity, improved sanitation, safe drinking water, clean cooking fuel, telephone and *pucca* (solid, permanent structure) houses was consistently higher in LMS than HMS in all NFHS survey rounds (figure 6). Yet in HMS, most indicators of household living conditions improved noticeably faster than in LMS during the 2005–2006 to 2019–2021 period, even closing the gap. The age at first cohabitation (after marriage) increased from a median of 17 years to 19 years between 2005–2006 and 2019–2021 in HMS, and from 18 to 19 years in LMS (data not shown). The proportion of literate women also improved faster in HMS than LMS, closing some of the gaps as HMS saw major increases in women’s literacy (1.5-fold), household electricity (by 2-fold), improved sanitation (3.2-fold) and telephone access (6-fold). In 2019, the HMSs were about 15 years behind the LMS for the proportion of households with *pucca* houses, use of clean fuel for cooking and women’s literacy.

### Health policy and systems drivers of MNH transitions in the state clusters

The state clusters with higher and lower mortality baselines also entered our period of analysis with markedly different health systems. Thus, they leveraged the national health policy and administrative changes since the 1990s—and particularly the NRHM since 2005—in different ways to improve universal access to health infrastructure and human resources. The NRHM, followed by the NHM, decentralised planning and increased state-level financial flexibility. States thereafter prepared annual project implementation plans (PIPs) that consolidated district-level data and health plans to articulate state-level problem assessments, goals and actions.

Commensurate with their lower baseline mortality levels, LMS’ generally strong foundations of governance, planning processes, data use and availability of infrastructure and human resources going into the NRHM allowed them to take immediate advantage of additional and more flexible funding for state-level initiatives through PIPs and related NRHM processes.

To me why NRHM was a big game changer [for Maharashtra was] because it allowed for the first-time lot of flexibility, which the state budgets were not doing basically…. Flexibility was provided by the Government of India both in terms of financial support and in flexibility of recruitment. (Maharashtra discussion group, expert #1, government)

When the central government offered more financing for MNH with the NRHM/NHM, LMS could quickly build on these opportunities to innovate and implement additional programmes: ‘the advantage with the low mortality states was they used to lap it up and, you know, implement it faster.’ (National KII 4, government technical and civil society)

Meanwhile, key informants expressed that HMS had previously relied heavily on directives from the central government, but the NRHM’s PIP process allowed them to systematically analyse their own data and set priorities, including need-based approaches for different districts. This expectation, combined with ‘handholding’ technical support from the centre and development partners, built these states’ capacity and ownership of health data and planning.

I think what happened with NHM was that states had to kind of pull up their shoes, and explain, why have I been [able] to spend the money, or not being able to spend the money. So, I think what happened, was, see, the push was never vertical. So, there were HR [human resource] initiatives, there were infrastructure initiatives, there were community initiatives, there was training of, you know, the set-up of the cadre, there were untied funds given to the sub-centres. Um, so I think, I think, what happened with this intense, kind of, reporting back, monitoring, etc. was that states started utilising the money better. (National KII 3, civil society)

State PIPs ‘galvanised’ action towards health outcome targets, including a ‘systematic, structured approach to develop plans for each geography’ (National KII 12, government technical and development partner).

Prior to that [the NRHM], you do not have a concept of even a state level plan with outcomes and so on. Starting from a base where the central government is looking for a plan where you [state governments] come back and commit to a certain level of MMR and so on. You never had that. So first getting to the state level and thinking in terms of outcomes itself was a big challenge. (National KII 5, government administrative & private sector)

The two state clusters then used the NRHM/NHM policies and PIP processes in somewhat different ways to improve the accessibility, availability, and later quality, of government health services. LMS started with overall higher availability of services and health workers prior to the NRHM. Thus, LMS could focus on improving access to ANC, delivery and PNC among disadvantaged groups specifically, and on improving quality of comprehensive EmONC facilities and special newborn care units (SNCUs) generally. This led to earlier increased deliveries at hospitals, and greater expansion of access to C-sections for the poorest. To do so, LMS also trained and certified many nurses and doctors, provided them further in-service training, and deployed specialists at more regional facilities targeting disadvantaged areas.

We have improved on the infrastructure, availability, accessibility, for all these cases have improved. Even if it is a tribal area all these facilities are available. When it comes to saving the life in case of obstetric emergencies, mainly being post-partum hemorrhage or maybe eclampsia, we have all the facilities available at the lower most level facility and we are able to save these women. (Maharashtra discussion group, expert #6, academic and technical)

LMS also developed medical service corporations early on to streamline procurement of free medicines even before the NRHM, and quickly focused on improving quality of health services through supervision, early adoption of health management information system monitoring systems, maternal death reviews, accreditation with the India Public Health Standards, and later built on these through the NHM’s LaQshya and Dakshata quality improvement programmes from 2017.

HMS focused on first establishing wider availability and access to basic MNH services, alongside emphasising the NRHM/NHM demand generation schemes including home visits by Accredited Social Health Activists (ASHAs) and universal Janani Suraksha Yojana (JSY) incentives.

Access was definitely an important point, increasing our delivery points, increase in points where C-sections facility was available. That was the basic thing […] areas where facilities were not there, creating facilities there. […] Community involvement, was through not only the Panchayati Raj structure but we had Village Health and Sanitation Committees, the ASHA worker… (Madhya Pradesh discussion group, expert #4, government)

Number one is system basics, that is system strengthening, infrastructure strengthening, increasing the number of health facilities, delivery points, FRUs [first referral units] etc. (Odisha discussion group, expert #2, government)

They expanded skilled birth attendance and basic EmONC at lower-level facilities through in-service training, skill and information technology (IT) labs, and supportive supervision for nurses and general doctors, as well as numerous supports to community health workers to enhance their performance.

Not many people may talk about it or even understand the enormity of it, but I think under the SBA [skilled birth attendant] initiative when tasks were shifted and the government allowed and trained ANMs [auxiliary nurse midwives] and staff nurses to do some of the skills, I think that was, that would have ended up in saving many, many lives. (National KII 3, civil society)

What was thought is, that if a staff doesn’t know how to examine the case, when to refer it, whether to take the delivery there only, or to refer it to the CHC [community health centre] or the District Hospital, or a big medical college—So the decision is very important here, at the grass root level. So, this training, Skilled Birth Attendant was started in all the districts. (Rajasthan discussion group, expert #1, government)

The state experts emphasised that the HMS established medical service corporations, and strengthened blood banking, emergency transportation, use of data systems and creating SNCUs in all districts hospitals over time as well.

## Discussion

This mixed-methods study sought to understand the exemplary trends in MMR and NMR reduction within two state clusters of India over the past three decades using the integrated mortality transition model.^7^ MMR and NMR levels in LMS were around 12 and 16 years ahead of HMS, respectively. If HMS continue the same pace of decline, they can also reach SDG targets for MMR and NMR by 2030 and enter early stage IV in 2035, as the LMS have already done. Since 2005, LMS continually reduced 0–2 days NMR, while HMS initially reduced 3–27 days NMR and more recently also reduced 0–2 days NMR. This was related to major declines in infectious diseases in HMS particularly, and later also in intrapartum conditions such as birth asphyxia. The latter improved greatly in LMS, with an increasing proportion of deaths now due to health status-related conditions (small vulnerable newborns, congenital anomalies or women’s anaemia) with declining mortality levels, as anticipated by the transition model.

While LMS started with higher and continually improving ANC, ANCq, institutional delivery and PNC, HMS accelerated coverage to start catching up. This is likely in part related to coverage increasing even more in the high priority districts of those states, which started with lower levels.^19^ C-sections were consistently high in LMS, often more than needed; C-sections increased somewhat but less acutely in HMS.^20^ Inequities in coverage were also reduced in both state clusters, though faster in LMS as found elsewhere.^21 22^ C-sections remained low among the poorest in HMS, but not LMS, in our study and others.^20^

Fertility declines occurred in both state clusters. Yet the number of births increased in HMS due to population momentum, thus increasing the burden on the health system, while births reduced in LMS, thus reducing the burden on the health system.^23^,^25^ However, lower fertility rates and increasing age at marriage resulted in fewer high-risk births in both state clusters, even more so in HMS than LMS. Other studies have linked this continuation of past trends to a combination of increasing women’s education, social norms shifting towards smaller family sizes, as well as higher contraceptive prevalence rates, particularly among socioeconomically disadvantaged groups among whom fertility had been highest.^25^,^27^ As socioeconomic development indicators improved in both clusters, the LMS continued to be about 15 years ahead, but HMS closed the gap particularly in household electricity, improved sanitation, telephone access and women’s literacy.^28 29^

Moving MMR and NMR levels down dramatically from mid-stage II to early stage IV of the transition, LMS made greater reductions in neonatal deaths on days 0–2, including those due to intrapartum complications. This was accompanied by increasing intervention coverage already from high levels, as well as showing evidence of more effective coverage of MNH interventions, by increasing coverage not only of ANC and institutional delivery, but also ANC with contents, hospital births and C-sections across socioeconomic groups. To do so, LMS quickly benefited from the NRHM’s administrative and financial flexibility, having a strong ability to use funds and data right away to inform decisions and target gaps. Other research shows that LMS started with higher service readiness, health worker density and proportion of first referral units (FRUs) with EmONC at earlier time periods and these continued to improve.^30^,^34^

To continue progressing to transition stage V, the transition model suggests LMS should prioritise health status related causes of death for neonates (small vulnerable newborns, congenital anomalies) and mothers (indirect causes such as anaemia). Further increases in antenatal care intensity, timeliness and contents (from almost 80% in 2017/2018) will be needed, particularly among left-out populations such as the poorest to approach universal coverage.^35^,^37^ With nearly universal institutional delivery, the current level of 71% hospital deliveries needs a further push for LMS to reach the median level of countries in transition stage V, and especially among the poorest groups. No great gains from fertility decline can further be expected in LMS (as further declines of fertility are unlikely and higher risk births have been largely eliminated); the LMS already have transition stage V fertility levels. Further socioeconomic improvements are needed to target the most deprived subpopulations, and to improve women’s and baby’s health status as much as possible.

At the same time, HMS reduced their mortality levels enough to shift from transition mid-stage I to early stage III since 2000. This was largely by decreasing late neonatal deaths on days 3–27 (mainly attributable to declines in infectious diseases), and more recently early neonatal deaths mainly due to intrapartum causes. In this period, HMS also greatly increased contact coverage by expanding ANC, and institutional deliveries particularly at lower level facilities that most often provide basic routine and emergency care. This study’s health policy and systems results indicate that to do so, HMS closely followed central guidelines and processes, and gained capacity to plan and use funding through the NRHM’s PIP mechanism to expand MNH services and simultaneously strengthen their health systems. These efforts were bolstered through the national designation of these states as Empowered Action Group states, and further defining high-priority districts within them, to afford them more financial and technical support.^1 19^ Our study also suggests that intervention coverage was improved through the JSY scheme and ASHA programmes even more so in HMS than LMS.^38^,^42^ By the NRHM, particular components of basic EmONC such as parenteral antibiotics, uterotonics and neonatal resuscitation were also being provided at lower level facilities (PHCs and CHC FRUs) in HMS.^43^ A more recent study found that health workers and EmONC at these facilities still remained lower in more of HMS’ than LMS’ districts in 2014.^44^ Unless quality is more widely improved, the FRUs’ life-saving role may reach a limit as deaths become increasingly concentrated in the intrapartum period.^45^

To further reduce maternal and neonatal mortality levels in the HMS beyond stage III, they can continue in the way of LMS by improving infection prevention while ramping up intrapartum care, and simultaneously building facility capacity to address prematurity and low birth weight. Substantial increases overall in ANC and PNC timeliness and contents are still needed in HMS.^35 37^ With NMR stagnant at home births, further gains can only be made by ensuring universal institutional delivery including among the poorer and rural populations. The LMS’ experience further indicates a need to put major emphasis on expanding hospital births compared with lower-level facility births, to improve access to more advanced care with C-sections and other life-saving EmONC interventions that can reduce days 0–2 deaths. Fertility declines in HMS are still possible, especially among the poorest, but the transition model indicates that related gains in mortality reduction are likely to be modest, as high-risk births have already reduced substantially. Further concerted efforts to harmonise policies across sectors would also continue to improve socioeconomic development in these states to ensure equitable progress.

This study used the MNH transition model as a novel tool to benchmark how state clusters have moved across different stages of mortality transition and indicate where they can go from here. We also aimed to understand the key health policy and systems drivers of state clusters’ transitions. The identified need to focus on improving MNH equity in both clusters aligns with India’s current push towards universal health coverage with its focus on leaving no one behind.^46^,^48^ The study aligns with others reasserting the need for states’ health systems to strengthen multisectoral partnerships to improve gender equity, living conditions and nutrition.^46^,^50^ This approach also highlights the value of comparing the respective health transitions of HMS and LMS over time from a strengths-based perspective, rather than comparing states’ higher and lower mortality levels and related factors at the same time point.^45 51^ The model can also aid in adapting lessons from other regions at similar or lower mortality levels in the past to pursue future gains in others.^52^

This study had some limitations. Data sources on maternal mortality causes and stillbirths were inadequate to include. Availability of comparable trend data on readiness and quality of services was limited. Health policy and systems findings developed from key informants and experts within group discussions could be subject to recall or social desirability bias, though a diverse sample across multiple sectors, pilot testing and member-checking likely helped ensure more trustworthy data.

## Conclusion

Our mixed-methods study analysed progress in five key characteristics of the maternal and neonatal mortality transition model in two distinct state clusters of India, and highlighted differential recommendations for continued improvement in these clusters. Overall, the LMS continued reducing MMR and NMR, particularly in the intrapartum period. These states successfully mobilised NRHM investments to build on the health system foundations already in place, further target efforts to close equity gaps, and ensure more deliveries at hospitals. Further gains could be made by focusing on increasing quality ANC and hospital births among the most disadvantaged groups, and improving nutrition to reduce health status related causes of death. For HMS, the NRHM’s decentralised financing and administrative processes helped strengthen their health system foundations, increase state-level ownership of health system data and planning, increase delivery points, expand emergency transport and provide in-service training and supportive supervision to health workers. HMS must further address intrapartum causes of death by expanding coverage across the continuum of care to the most disadvantaged groups and hospital and C-section deliveries generally, while improving socioeconomic conditions to improve women’s and newborn’s health and nutritional statuses. The study found the maternal and neonatal mortality transition model highly valuable to characterise where states have come from and consider critical steps to further improve maternal and newborn survival.

## Supplementary material

10.1136/bmjgh-2022-011413 online supplemental file 1

10.1136/bmjgh-2022-011413 online supplemental file 2

## Data Availability

Data are available on reasonable request.
