# Supplementary material for: A tale of two exemplars: the maternal and newborn mortality transitions of two state clusters in India
Source: BMJ Glob Health. 2024 May 2;9(Suppl 2):e011413. doi: 10.1136/bmjgh-2022-011413 (PMC11085921; doi:10.1136/bmjgh-2022-011413)
Supplement: online supplemental file 1 [file bmjgh-2022-011413supp001.docx]

## Supplementary materials 1

Table S1: Maternal and neonatal mortality transition model stages

| **Stage** | **Mortality levels** | | **Cause of death patterns** | **Intervention coverage/equity** | **Fertility/high risk births** | **Socio-economic development** |
| --- | --- | --- | --- | --- | --- | --- |
|  | *MMR, per 100,000 live births* | *NMR, per 1000 live births* |  |  |  |  |
| **I** | 700+ | 45+ | Infectious diseases most common cause of death | Extremely low, except among well-off groups (top inequalities), with lower service availability/access | High total fertility rate levels (~ 6-7) | Lower socio-economic conditions and female education |
| **II** | 300-700 | 30-45 | Infectious diseases and peri-partum conditions cause fewer deaths, increasingly due to causes related to health status of mother/ baby | Increasing contact coverage for basic services, and increasingly more advanced and with quality, moving to linear then bottom inequalities (worst-off lack access) | Fertility levels continually drop (between 5 and 2), while smaller proportion of high-risk births (low/high maternal age, high parity, short birth interval) | Improving socio-economic conditions, with large improvements between stages II and III in women’s education levels |
| **III** | 100-300 | 15-30 |  |  |  |  |
| **IV** | 20-100 | 5-15 |  |  |  |  |
| **V** | <20 | <5 | Near to all preventable deaths eliminated | Near to universal access to high-quality care, more at hospitals, with emergency obstetric and newborn care (EmONC) | Fertility low (below 2) with only unavoidable risk births (primiparous mothers over 18) | Good socio-economic conditions and high women’s education levels |


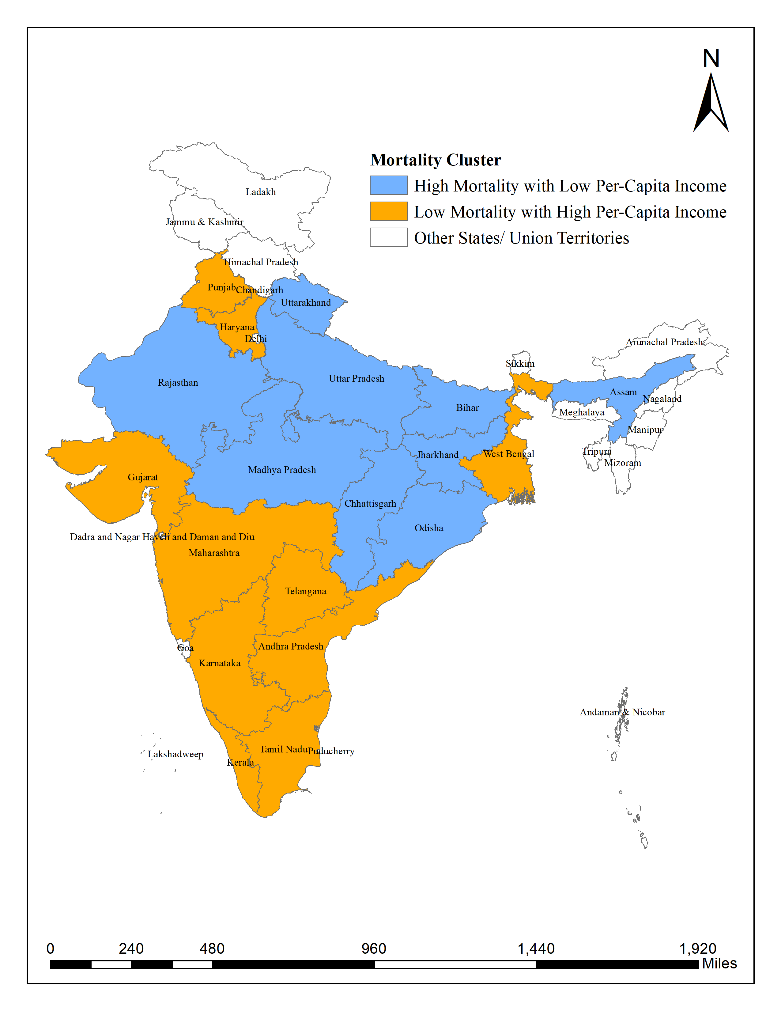


Figure S1: Map of India showing high and low mortality state clusters


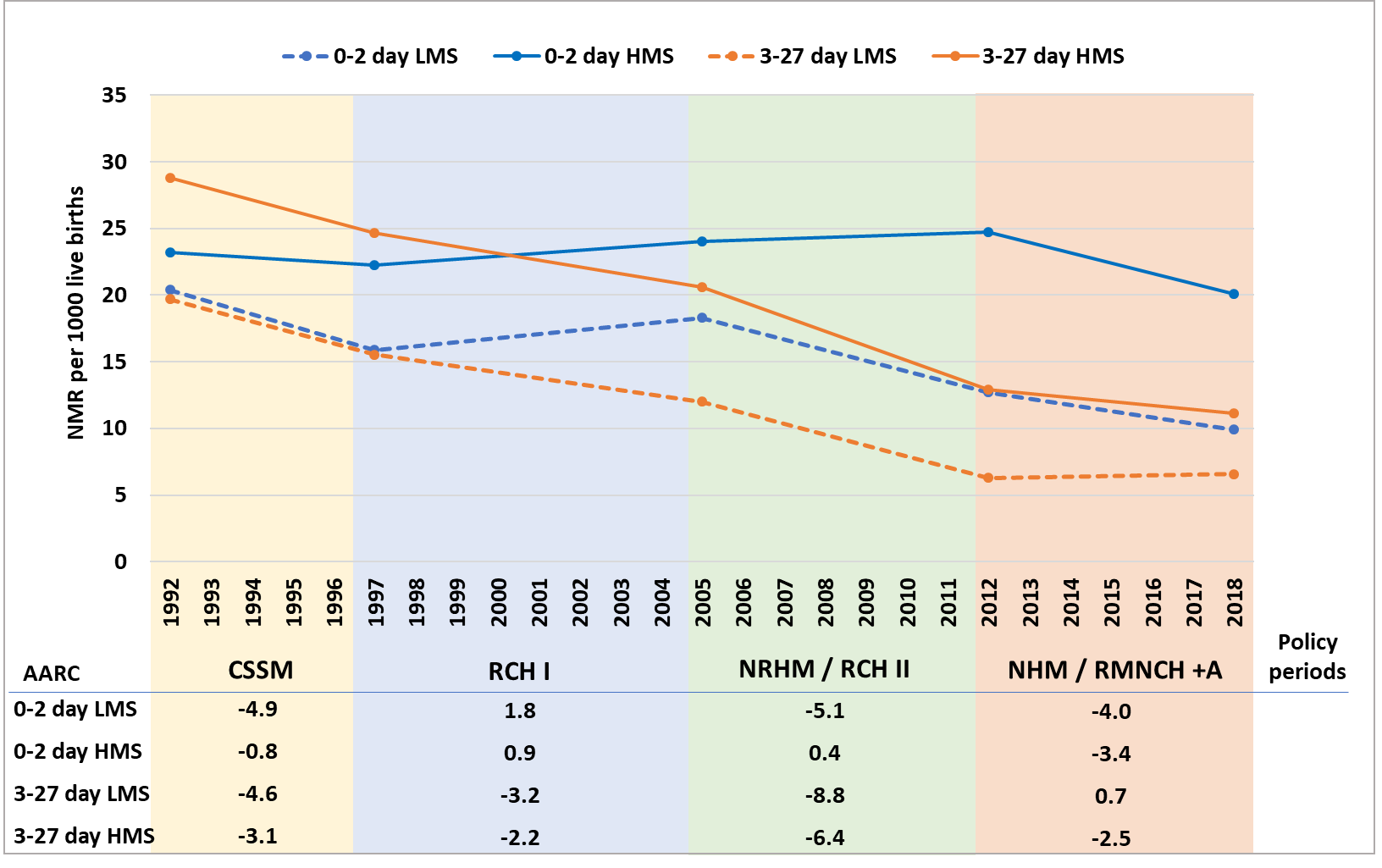
Figure S2: Neonatal mortality per 1000 live births at days 0-2 and 3-27, and the average annual rates of change (AARC, %) in different policy periods, in lower and higher mortality state clusters (NFHS 1992-3, 1998-99, 2005-6, 2015-16 and 2019-21)

Figure S3: Percent distribution of neonatal deaths by major causes of death in the LMS and HMS, MDS, WHO-MCEE, and GBD, 2000-19


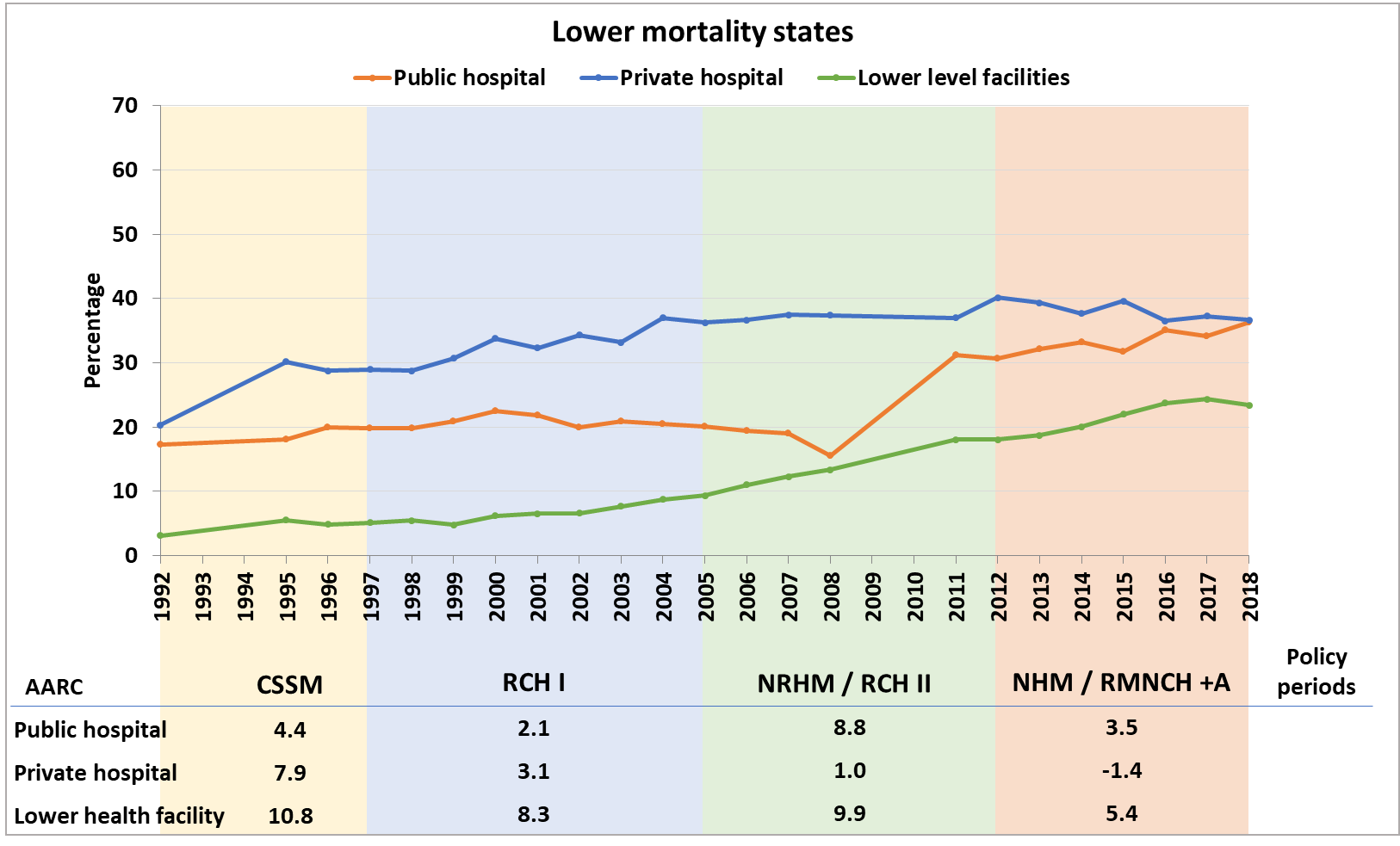


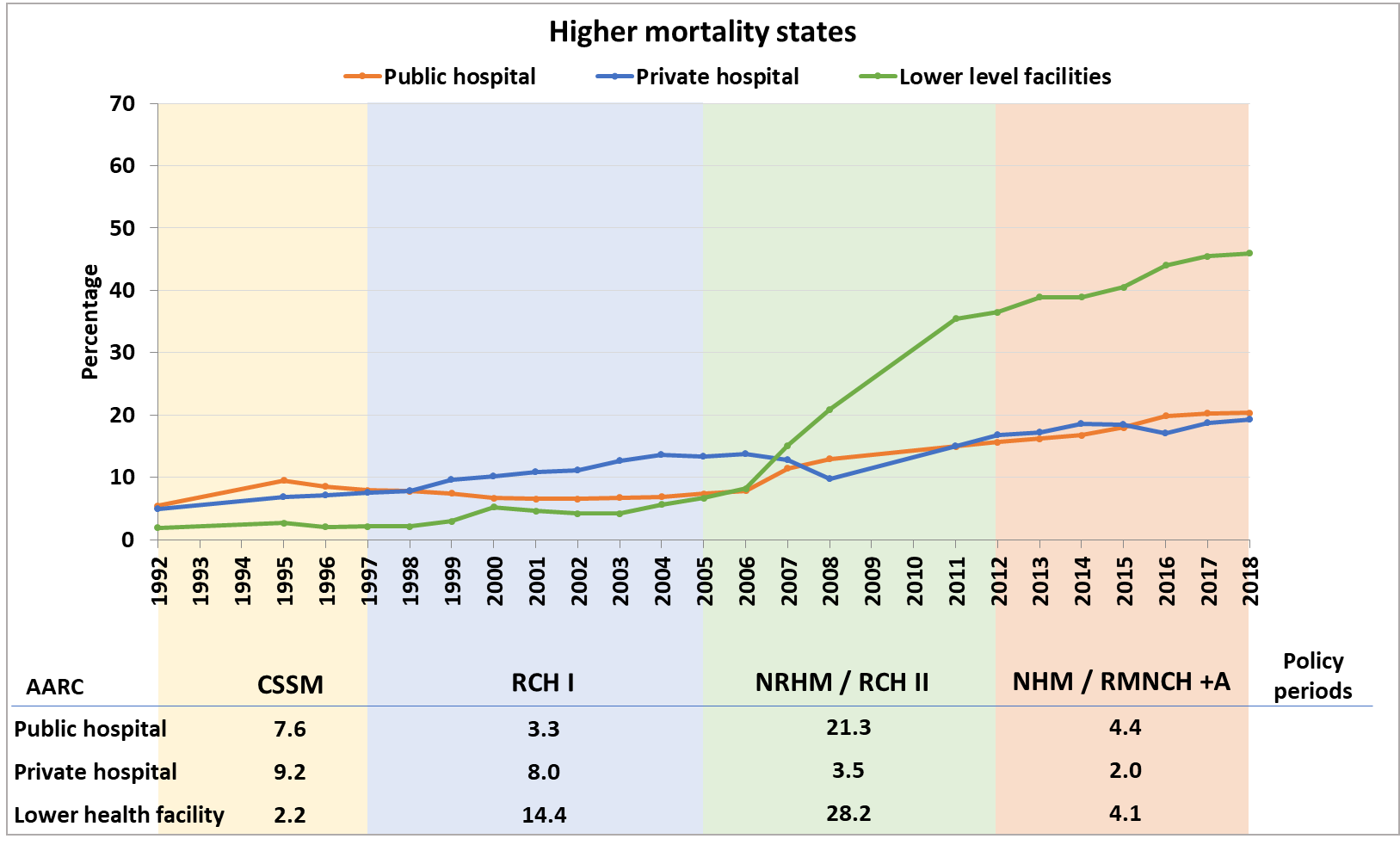
Figure S4: Trends in institutional delivery by facility type in the lower and higher mortality state clusters, India (NFHS and DLHS pooled data, 1992-2018)


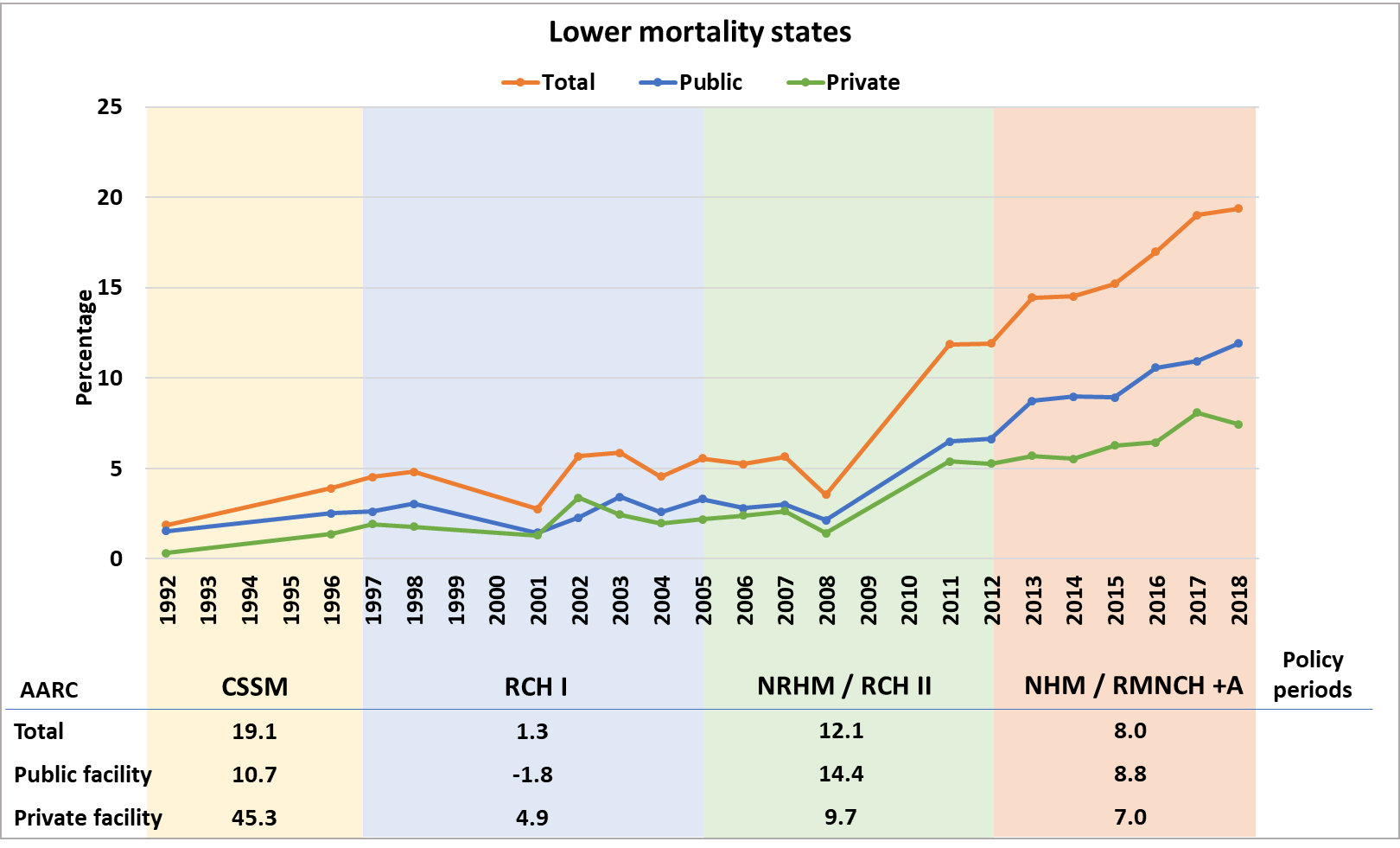


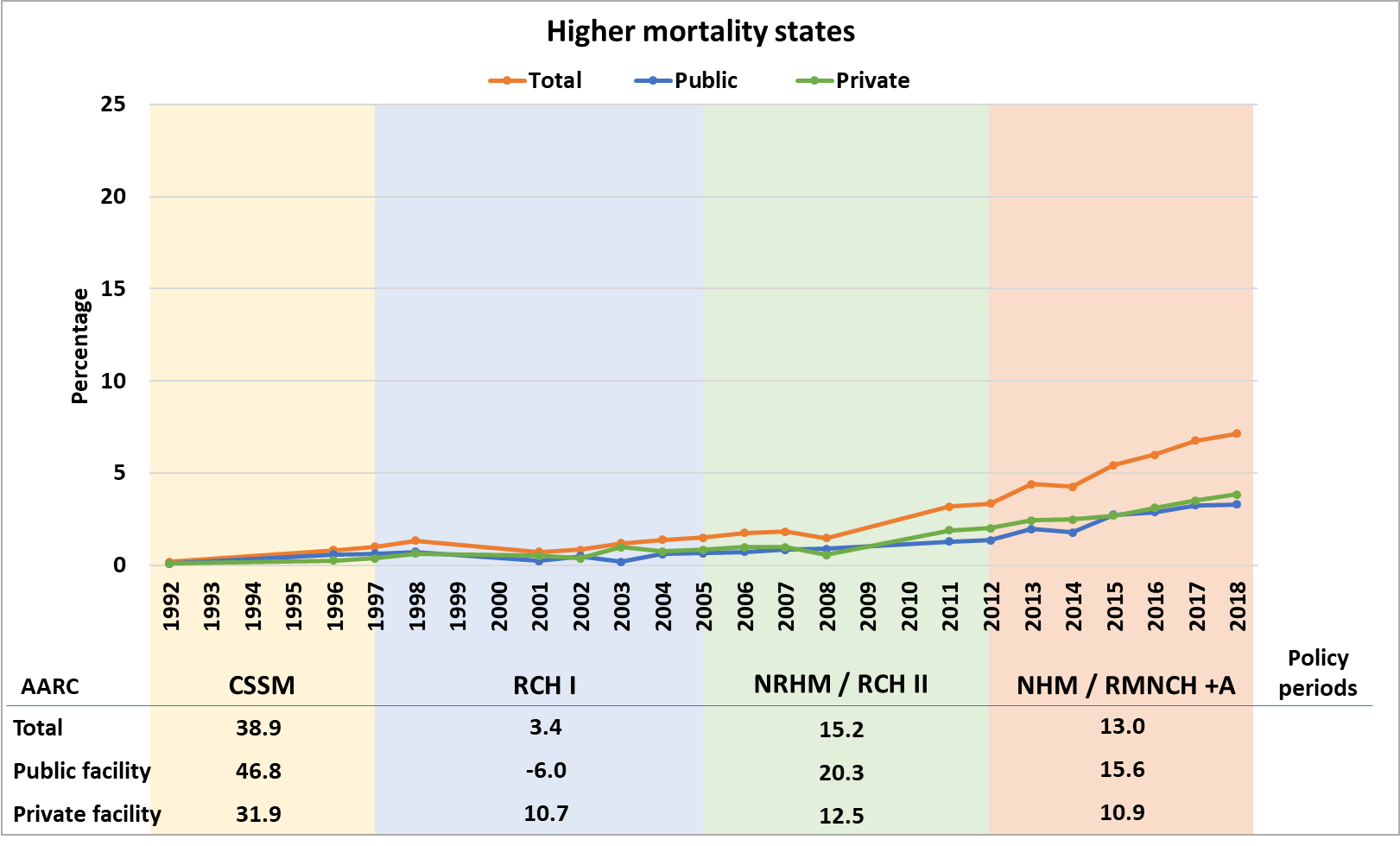


Figure S5: Trends in C-section rates among the poorest wealth quintile by facility type and state cluster, India (NFHS and DLHS pooled data, 1989-2018)

Figure S6: Trends in total fertility rate in higher and lower mortality state clusters (SRS, 1970-2019)

Figure S7: Trends in the percentage of children born in the 5 years preceding the survey in single, multiple and no high-risk birth category, in higher and lower mortality states (NFHS 2005-6, 2019-21)

Table S2: Distribution of maternal and newborn deaths saved due to fertility change and contribution of fertility change to observed decline in MMR and NMR in the two state clusters (Jain method of decomposition of effects of fertility on MMR and NMR)

|  | Distribution of deaths saved due to fertility change and safe motherhood | | | | Contribution of fertility change to observed decline | | | |
| --- | --- | --- | --- | --- | --- | --- | --- | --- |
|  | Maternal | | Newborn | | Maternal mortality ratio | | Neonatal mortality rate | |
|  | HMS | LMS | HMS | LMS | HMS | LMS | HMS | LMS |
| Due to fertility decline | 12 | 14 | 27 | 19 | 29 | 30 | 29 | 30 |
| Due to safe motherhood programs | 63 | 60 | 52 | 57 | 71 | 70 | 71 | 70 |
| Due to overlap of both (age-parity changes) | 25 | 26 | 22 | 24 |  |  |  |  |
