# Supplementary material for: A tale of two exemplars: the maternal and newborn mortality transitions of two state clusters in India
Source: BMJ Glob Health. 2024 May 2;9(Suppl 2):e011413. doi: 10.1136/bmjgh-2022-011413 (PMC11085921; doi:10.1136/bmjgh-2022-011413)
Supplement: online supplemental file 2 [file bmjgh-2022-011413supp002.pdf]

Supplementary materials 2: Reflexivity Statement

1. How does this study address local research and policy priorities?

The focus of this study aligns with the interests and priorities of the National Health Systems Resource Centre (NHSRC), which is the Government of India's Technical Support Institute for the National Health Mission. NHSRC coordinated and led the study in collaboration with the International Institute for Population Sciences (Mumbai), supported by the India Health Action Trust (IHAT) (New Delhi) and the University of Manitoba (UM), Canada.

2. How were local researchers involved in study design?

The study was led by researchers in India and all partner organization were involved in the finalization of research design. An Expert Working Group, constituted by the Ministry of Health and Family Welfare, Government of India, provided technical inputs on the study design. All mixed-methods analyses, integration and interpretation of findings were jointly conducted by researchers from NHSRC (focusing on the health policy and systems analysis) and IIPS (leading the quantitative analysis) with support from India Health Action Trust and the University of Manitoba at each stage.

3. How has funding been used to support the local research team?

This project funded six mid-career full-time research fellowships and partially supported senior researchers' time at the lead organizations in India.

4. How are research staff who conducted data collection acknowledged?

This analysis leveraged existing quantitative datasets, thus requiring no primary quantitative data collection. The qualitative primary data collectors are acknowledged as co-authors (HB, KS, AKB, BMR, UR, RW) and in the study team (MR, TN & DD).

5. Do all members of the research partnership have access to study data?

All members of the partnership have access to data.

6. How was data used to develop analytical skills within the partnership?

IIPS (Mumbai) built on their existing expertise in quantitative analysis of health surveys, and NHSRC on their expertise in health policy and systems strengthening. The study provided opportunities for research fellows at these two organizations to develop further analytic skills in analysis of multiple rounds of national datasets using Excel and Stata, as well as qualitative analysis in Dedoose, respectively.

7. How have research partners collaborated in interpreting study data?

The study team has met biweekly since the inception of the study to plan the study design, data collection, analysis, interpretation and dissemination. We also held national and state-level stakeholder meetings that sought inputs from experts on the findings to refine interpretations.

8. **How were research partners supported to develop writing skills?**

The research partners worked jointly on numerous national and international oral presentations and written outputs, allowing mutual support and opportunities to build further writing and communication skills using different formats.

9. **How will research products be shared to address local needs?**

The study's reports, academic papers and presentations are being shared in national and international fora to convey its key learnings on how India improved maternal and newborn survival in the past two decades, as well as providing a framework for understanding how improvements can further be made. This aligns with India's National Health Mission priorities and ethos of learning from the past to inform the future. Dissemination activities in India included presentations at the following events to date:

- The 2021 International Population Conference, organized by IUSSP (International Union for the Scientific Study of Population), in Hyderabad in December 2021
- The Distinguished Lecture Series organized by the Indian Association for the Study of Population (IASP) in October 2022
- The 43rd Annual Conference of the Indian Association for the Study of Population (IASP) on 2 December, 2022
- The IIPS International Seminar in Bengaluru in March 2023

10. **How is the leadership, contribution and ownership of this work by LMIC researchers recognised within the authorship?**

Authors HB and UR are first/last authors as the principal investigators of the study in India. The authorship also includes their team members from NHSRC and IIPS, as well as IHAT and UM, who worked together on the study including the writing of this manuscript.

11. **How have early career researchers across the partnership been included within the authorship team?**

Early career researchers have been involved in this partnership from both NHSRC (AK, DD, MR) and IIPS (PK, KA, SG).

12. **How has gender balance been addressed within the authorship?**

Four main authors (UR, KS, AB, RA) are women and four main authors (HB, PK, BMR, RW) are men.

13. **How has the project contributed to training of LMIC researchers?**

The study team was led by researchers in India, and as an interdisciplinary team the study allowed mutual learning and training opportunities. The project also provided funding for the research fellows within India to continue professional development and training in their respective fields.

14. How has the project contributed to improvements in local infrastructure?

This project has not directly contributed to improvements in local infrastructure, but has developed long-term research partnerships between study partners that are continuing beyond the specific project.

15. What safeguarding procedures were used to protect local study participants and researchers?

The study was conducted in accordance with ethics approval from the IIPS and UM ethics review boards. Data with participants was collected confidentially and were de-identified in the results. Researchers gained support for and input on the study design based on the local priorities of the government. We then spent significant time meeting twice monthly to discuss, plan and reflect on the study activities. This helped us develop strong relationships of trust and open communication, which promoted equitable and open decision-making around the processes and outcomes of the study, as well as ensuring the results and dissemination were contextually-sensitive and applicable.
